# Exploring Fingerprint Biometric Devices as a Source of Multidrug Resistant Bacterial Isolates in a Tertiary Care Hospital

**DOI:** 10.4314/ejhs.v36i1.2

**Published:** 2026-01

**Authors:** Sooraj Ahuja, Kxitiza Pandey, Sulekha Nautiyal, Dimple Raina

**Affiliations:** 1 Department of Microbiology, Shri Guru Ram Rai Institute of Medical and Health Sciences, Patelnagar, Dehradun. Pin Code: 248002

**Keywords:** Fingerprint biometric devices, Nosocomial infection, Hospital-acquired infections (HAIs), Hand hygiene

## Abstract

**Background:**

Biometric fingerprint systems, although effective for identification and security, can facilitate the spread of infections in hospital settings due to repeated contact by multiple users. Hospital-acquired infections (HAIs) are increasingly associated with bacteria such as Staphylococcus aureus, Acinetobacter spp., Escherichia coli, and Pseudomonas species, which are capable of surviving on inanimate surfaces, including fingerprint scanners. This study was conducted to determine whether biometric fingerprint devices can act as reservoirs for pathogenic microorganisms and to detect the presence of multidrug-resistant organisms (MDROs) along with their antibiotic susceptibility profiles.

**Methods:**

Samples were collected from biometric fingerprint devices installed in the hospital and medical college. The samples were cultured, and the isolated organisms were identified. Antibiotic susceptibility testing of the isolates was performed using an automated system (VITEK 2 Compact, bioMérieux).

**Results:**

The overall prevalence of pathogenic bacteria isolated was 17.7% (17/96). Among the pathogenic bacteria identified (n=17), 29.4% were Gram-positive cocci, while 70.6% were Gramnegative bacilli. The most common bacteria isolated were Serratia species (23.5%), followed by Acinetobacter species and Enterococcus species. Among Gram-positive cocci, Enterococcus species were the most frequently isolated organisms.

**Conclusion:**

In conclusion, fingerprint biometric devices may act as reservoirs for multidrug-resistant organisms and contribute to cross-transmission in hospitals. Contactless biometric devices are preferable; otherwise, regular disinfection, nearby hand sanitizers, and routine microbiological surveillance should be part of infection control audits.

## Introduction

The term “biometric” refers to the measurement and analysis of unique physical and behavioral characteristics of individuals. It is commonly associated with identification, verification, and security systems, where biometric data such as fingerprints, facial recognition, iris scans, and voice patterns are used to establish identity (1). Fingerprint biometric systems identify individuals through direct contact between the fingertip skin and the device surface, providing a secure and efficient method of identification. Consequently, institutions such as offices, hospitals, and government agencies increasingly use biometric identification systems for attendance monitoring. Most hospitals currently employ fingerprint biometric devices to record attendance of healthcare workers, including doctors, nurses, ward attendants, and housekeeping staff. However, these devices can act as potential sources of transmission for pathogenic microorganisms. Hospital-acquired infections (HAIs), also referred to as nosocomial infections, continue to increase and are associated with significant morbidity and mortality. These infections are acquired from the hospital environment (2). Healthcare workers may inadvertently transmit microorganisms such as *Staphylococcus aureus, Pseudomonas aeruginosa, and Acinetobacter baumannii*, including their drug-resistant forms such as *methicillin-resistant Staphylococcus aureus (MRSA), vancomycin-resistant Enterococcus (VRE)*, and multidrug-resistant Gram-negative bacteria. These pathogens can survive on dry surfaces for prolonged periods, increasing the risk of transmission (3).

Several studies have demonstrated that medical devices such as thermometers, stethoscopes, and aprons can harbor microorganisms (4–7). Even objects not traditionally associated with healthcare, such as keyboard covers and ballpoint pens, have been identified as potential sources of infection (8).

Microorganisms can also form biofilms on solid surfaces. Biofilm-associated bacteria exhibit substantially greater resistance to antimicrobial agents compared to their planktonic counterparts. This increased resistance poses a heightened risk when such bacteria contaminate biometric devices and other fomites. Although regular disinfection of fingerprint scanners is often recommended, this approach may be impractical due to the resilience of biofilm-forming bacteria and the potential for disinfectants to further promote antimicrobial resistance (9). As a precautionary measure, many hospitals have installed hand sanitizer dispensers near biometric devices to address employee safety concerns (10).

This study was undertaken to detect the presence of multidrug-resistant bacterial strains on biometric fingerprint devices installed in a healthcare setting with continuous exposure to infectious agents and to assess the role of fingerprint-based biometric systems as potential sources of MDROs.

## Materials and Methods

This prospective cross-sectional study was conducted by the Department of Microbiology at Shri Guru Ram Rai Institute of Medical and Health Sciences, Dehradun, and the associated Shri Mahant Indiresh Hospital. The study was carried out over a period of two months after obtaining approval from the Institutional Research and Ethical Committee (letter no. SGRR/IEC/01/23).

A total of 12 biometric fingerprint devices installed at different locations within the hospital and medical college and routinely used by healthcare workers were included in the study. Each device was assigned a location code, and swabs were collected once weekly on a fixed day at 4:00 PM (Indian Standard Time) for two months. The devices were not disinfected prior to sample collection.

Samples were collected using sterile cotton swabs (PW003, HiMedia) moistened with brain heart infusion (BHI) broth (M210, HiMedia). The swabs were rotated over the fingerprint surface for 5–10 seconds and then placed in tubes containing BHI broth. Samples were transported promptly to the microbiology laboratory after appropriate labeling with site, date, and time of collection. All swabs were processed individually without undue delay.

The specimens in BHI broth were incubated at 37°C for six hours and subsequently inoculated onto 5% sheep blood agar (BA 43041, bioMérieux) and MacConkey agar (M082, HiMedia). Culture plates were incubated aerobically at 37°C for 16–18 hours. Grown colonies were examined using Gram staining to identify Gram-positive cocci and Gram-negative bacilli. Gram staining was also used to exclude *Bacillus species* (except *Bacillus anthracis*) and diphtheroids, which were considered probable skin contaminants in the hospital setting. The isolates were further tested using catalase, coagulase, and oxidase tests. Final identification and antimicrobial susceptibility testing were performed using an automated system (VITEK 2 Compact, bioMérieux).

The results were entered into a Microsoft Excel spreadsheet, and numerical variables were analyzed using counts and percentages.

Ethical approval for the study was obtained from the Institutional Research and Ethical Committee (Letter No. SGRR/IEC/01/23).

## Results

A total of 96 swab specimens were collected from the fingerprint contact surfaces of 12 biometric devices installed at various locations, including the medical college, administration block, and hospital. Of these, 79 swabs showed growth of *Bacillus species* and diphtheroids *(Corynebacterium species)*, which were considered probable skin contaminants and were excluded from further analysis. The remaining 17 swabs yielded growth of suspected pathogenic bacteria (Gram-positive cocci or Gram-negative bacilli) and were subjected to biochemical identification and antimicrobial susceptibility testing.

Among the 17 pathogenic isolates, two were obtained from biometric devices in the medical college, one from the administration block, and 14 from devices installed at various hospital locations, including the Medical Superintendent Office, Nursing Superintendent Office, Human Resource Office, Cardiology OPD, and Main OPD Block. Only devices routinely used by both doctors and nursing staff were included.

The overall prevalence of pathogenic bacteria was 17/96 (17.7%). Of the pathogenic isolates, 5/17 (29.4%) were Gram-positive cocci and 12/17 (70.6%) were Gram-negative bacilli. The most common Gram-positive cocci isolated were *Enterococcus species* (Table 1). Among Gram-negative bacilli, *Serratia species* were the most frequently isolated, followed by *Acinetobacter species* (Table 1). Of the four *Serratia* isolates, three were obtained from hospital-based biometric devices and one from the medical college.

**Table 1:** Distribution of bacterial isolates (n=17)

Bacterial group	Organisms isolated	Number (%)
Gram	*CONS*	2 (11.8)
positive	*Enterococcus faecium*	1 (5.9)
cocci (n=5)	*Enterococcus faecalis*	2 (11.8)

Gram	*Acinetobacter baumanii*	1 (5.9)
negative	*Acinetobacter lwofii*	2 (11.8)
bacilli	*Aeromonas salmonicida*	1 (5.9)
(n=12)	*Enterobacter cloacae*	1 (5.9)
	*Pseudomonas aeruginosa*	1 (5.9)
	*Serratia marcescens*	3 (17.6)
	*Serratia odorifera*	1 (5.9)
	*Sphingomonas*	2 (11.8)
	*paucimobilis*	

**Fig 1:**
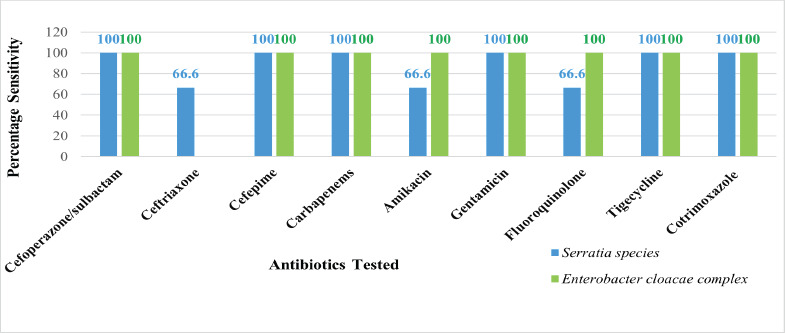
Antimicrobial Susceptibility pattern of Enterobacterales isolated

All members of the family *Enterobacterales* demonstrated sensitivity to most of the antibiotics tested (Figure 1).

**Fig 2:**
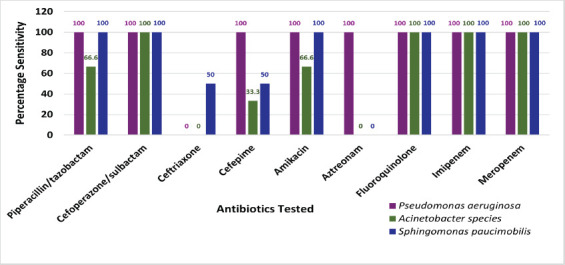
Antimicrobial susceptibility pattern of non-fermenters isolated in the study

Among the non-fermenters isolated (6/17; 41.17%), *Pseudomonas aeruginosa* (16.67%), *Acinetobacter species* (50%), and *Sphingomonas paucimobilis* (33.33%) were evaluated for antimicrobial susceptibility. Resistance to ceftriaxone was observed in all isolates of *Pseudomonas aeruginosa* and *Acinetobacter species*, while resistance to aztreonam was noted in all isolates of *Acinetobacter species* and *Sphingomonas paucimobilis* (Figure 2).

All three *Enterococcus* isolates were sensitive to most antibiotics tested. However, both isolates of *coagulase-negative Staphylococcus species* were found to be methicillin resistant

## Discussion

Touch-based biometric fingerprint devices are widely used in hospitals to monitor healthcare workers' attendance. However, repeated contact by multiple users can lead to microbial colonization and facilitate the spread of multidrug-resistant pathogens through cross-contamination. Fomites are well-recognized reservoirs of pathogens, and inadequate hand hygiene practices among healthcare workers can contribute to contamination of these devices. In the post–COVID-19 era, although the importance of hand hygiene has been strongly emphasized, concerns regarding the safety of shared touch-based equipment persist (11). This study was undertaken to evaluate whether biometric fingerprint devices can act as reservoirs for MDROs in the hospital environment.

The study was conducted in a 1200-bedded tertiary care hospital located in an urban setting, where healthcare workers operate on a shift basis. Of the 96 swabs collected from 12 biometric devices, pathogenic bacteria were isolated from 17.7% of samples. Among these isolates, 11.8% were from the medical college, 5.9% from the administration block, and 82.3% from biometric devices installed at various hospital locations. Similar findings were reported by Jogender et al. (2019), who observed contamination rates of 13.33% in administrative blocks, 26.67% in medical colleges, and 60% in hospital settings (12).

Previous studies have reported the presence of *MRSA, Klebsiella pneumoniae, Acinetobacter baumannii*, and *Enterococcus faecalis* on stethoscopes and other inanimate hospital objects (4–8). Contamination of biometric devices in the administrative block and medical college may be attributed to healthcare workers moving between hospital and academic settings and using biometric devices at multiple locations.

The organisms isolated in the present study included both Gram-positive cocci and Gram-negative bacilli. The most common pathogens identified were *Serratia species, Acinetobacter species, Enterococcus species, coagulase-negative Staphylococcus species*, and *Sphingomonas paucimobilis*. These organisms are well-established causes of nosocomial infections and are associated with high morbidity and mortality due to their intrinsic or acquired multidrug resistance. Their presence on biometric fingerprint devices highlights the potential role of these devices in the transmission of HAIs (13). Although most isolates demonstrated susceptibility to multiple antibiotics (Fig. 3 and Fig. 4), the isolation of *methicillin-resistant coagulase-negative Staphylococcus species* is concerning.

Intrinsic antimicrobial resistance plays a crucial role in determining appropriate treatment strategies (14). Even when isolates exhibit overall sensitivity, knowledge of their resistance patterns is valuable during outbreak investigations. To minimize the risk of transmission, contactless biometric systems such as facial recognition or iris scanning may be considered, along with strict adherence to hand hygiene practices (15).

This study indirectly reflects the level of hand hygiene compliance among healthcare workers. Limitations include a small sample size and the exclusion of anaerobic and fungal pathogens. Larger, more comprehensive studies are warranted. Given the limited number of Indian studies addressing the contamination potential of fingerprint biometric devices, the findings of this study are particularly relevant.

In conclusion, this study demonstrates that fingerprint biometric devices can harbor a variety of bacterial contaminants, including multidrug-resistant isolates. These findings indicate that such commonly used devices may act as reservoirs for healthcare-associated pathogens and contribute to cross-transmission within hospital settings. The use of alternative contactless biometric systems, such as facial recognition or iris scanning, is recommended. Where fingerprint devices are used, regular disinfection of device surfaces and placement of hand sanitizers adjacent to these devices are essential. Additionally, routine bacteriological surveillance of biometric fingerprint scanners should be incorporated into periodic hospital infection control audits.
